# Supplementary material for: Doubled strength and ductility via maraging effect and dynamic precipitate transformation in ultrastrong medium-entropy alloy
Source: Nat Commun. 2023 Jan 10;14:145. doi: 10.1038/s41467-023-35863-z (PMC9832006; doi:10.1038/s41467-023-35863-z)
Supplement: Supplementary file 1 — Supplementary information [file 41467_2023_35863_MOESM1_ESM.pdf]

Supplementary information for

Doubled strength and ductility via maraging effect and dynamic precipitate transformation in ultrastrong medium-entropy alloy

Hyun Chung [1,†], Won Seok Choi [2,†], Hosun Jun [2], Hyeon-Seok Do [3], Byeong-Joo Lee [3], Pyuck-Pa Choi [2], Heung Nam Han [4], Won-Seok Ko [5], Seok Su Sohn [1,*]

[1] Department of Materials Science and Engineering

Korea University, 02841 Seoul, South Korea

[2] Department of Materials Science and Engineering

Korea Advanced Institute of Science and Technology, 34141 Daejeon, South Korea

[3] Department of Materials Science and Engineering

Pohang University of Science and Technology, 37673 Pohang, South Korea

[4] Department of Materials Science and Engineering

Seoul National University, 08826 Seoul, South Korea

[5] Department of Materials Science and Engineering

Inha University, 22212 Incheon, South Korea

[†]These authors contributed equally.

* Corresponding authors: sssohn@korea.ac.kr

Supplementary Note. Details of phase stability calculations The present DFT calculations were performed to examine the phase stabilities of ordered precipitates and disordered matrix at finite temperatures. It is impractical to construct a multi-dimensional convex hull of the free energy because the types and compositions of related solid solutions and compounds to be considered in the target ternary system are too vast to be dealt with by DFT calculations. Therefore, predicting the phase equilibrium of the alloy at finite temperature was performed with additional thermodynamic calculations. Instead, the present calculation aimed to provide information about the possible metastability of ordered precipitates in the following sense. According to previous investigations based on Calphad-type thermodynamic calculations[1,2], the first precipitate that is most likely to nucleate is the one with the largest driving force for the nucleation, which is defined by the free energy difference between the parent phase and the precipitate phase at a certain composition. The present DFT calculation provided driving forces of candidate precipitates in different compositions by comparing their relative stabilities with the corresponding bcc solid solution of the same composition. We expected that the precipitate of a composition with the largest driving force is more likely to be present at least at the initial stage of the ageing treatment even though there is a possibility of succeeding phase transformation through the over-ageing if equilibrium phases are differently presented.

To investigate the physical properties of an ordered structure, a possible distribution of each constituting element in sublattice sites should be decided first. The reported stoichiometry (A_3B_1-type) of the stable compound (hP24-Co_3V_1) in the Co–V binary system[3–6] and the experimentally obtained composition ($Co_{\sim0.5}V_{\sim0.25}Fe_{\sim0.25}$) of the ordered structure (hP24 and $L1_2$) imply that one of the elements of V and Fe share the same sublattice site with Co (75%) and the other element occupies the other sublattice (25%). For example, there is a possibility

of partial disordering of Co and Fe atoms at the same sublattice ($(Co,Fe)_3V_1$) or partial disordering of Co and V atoms at the same sublattice site ($(Co,V)_3Fe_1$). To clarify this issue, we compared the formation energies of binary ordered structures with different occupations of the second sublattice by V and Fe (Co_3V_1 vs. Co_3Fe_1) as listed in Supplementary Table 3. The obtained difference in the formation energies of ordered structures, e.g., $L1_2$-Co_3V_1 structure (–0.166 eV/atom) and $L1_2$-Co_3Fe_1 structure (0.013 eV/atom), implies that the sublattice of $(Co,Fe)_3V_1$ is much more stable and feasible state compared to the sublattice of $(Co,V)_3Fe_1$.

We thus considered the partial disordering of Co and Fe atoms in the same sublattice site (e.g., $L1_2$-$(Co,Fe)_3V_1$) for the generation of SQS cells for ordered structures. For all candidate precipitates analogous to the close-packed fcc and hcp structures ($L1_2$, hP24, and $D0_{19}$), different populations of the (Co,Fe) lattice were considered, with a Co concentration in the sublattice ranging from 0 to 100% (Co_3V_1, $Co_2Fe_1V_1$, $Co_{1.5}Fe_{1.5}V_1$, $Co_1Fe_2V_1$, and Fe_3V_1). For the bcc solid solution, we considered the complete disordering of all elements. To improve expectations on the disordered state, five different SQS supercells with 48 atoms were independently prepared using different correlation functions (pair and triple) within different ranges. The phase stability was then predicted by averaging obtained values of independent runs for different SQS supercells.

Mechanical property details of other HEAs. The data sources for the different alloys plotted in Fig. 3c are for fcc single-phase HEAs[7–10], refractory bcc single-phase HEAs[11–14], bcc/hcp dual-phase HEAs[12], fcc/bcc dual-phase HEAs[15–19], fcc/hcp dual-phase HEAs[20], fcc HEAs with intermetallic compounds[8,9,21–25], bcc HEAs with intermetallic compounds[26], and maraging steels[27–31].

Determination of dislocation densities through KAM approach. The kernel average misorientation (KAM) was calculated up to the third neighbour shell with a maximum orientation angle of 5°. The geometrically necessary dislocations (GNDs) were determined from the KAM values using Eq. (1)[32]:

$$\rho_{\mathrm{GND}}\ (m^{-2}) = \frac{2\theta}{ub} \quad (1)$$

where u is the unit length, b is the magnitude of Burgers vector, and θ is the misorientation.

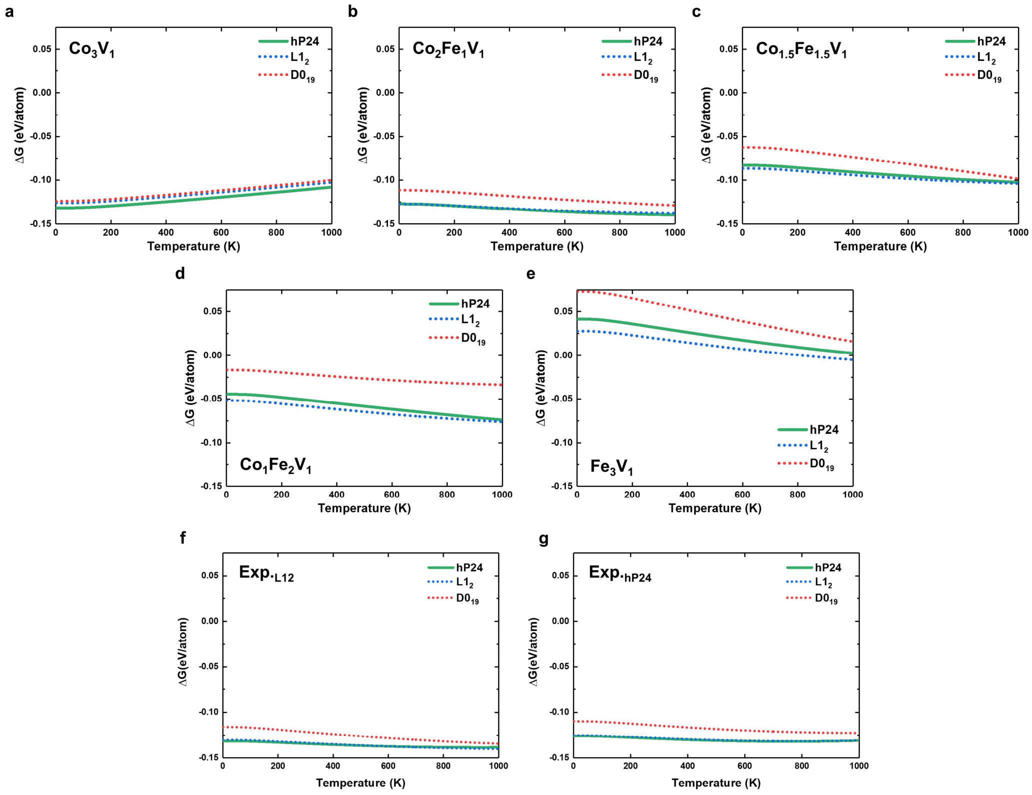

Supplementary Fig. 1. Temperature dependence of the Gibbs energy of candidate ordered precipitates (hP24, $L1_2$, and $D0_{19}$) with respect to the disordered bcc solid solution approximated based on the Debye–Grüneisen model. The results for different compositions (**a** Co_3V_1, **b** $Co_2Fe_1V_1$, **c** $Co_{1.5}Fe_{1.5}V_1$, **d** $Co_1Fe_2V_1$, **e** Fe_3V_1, **f,g** experimentally obtained composition of the precipitates provided in Supplementary Table 1 and 4) are presented.

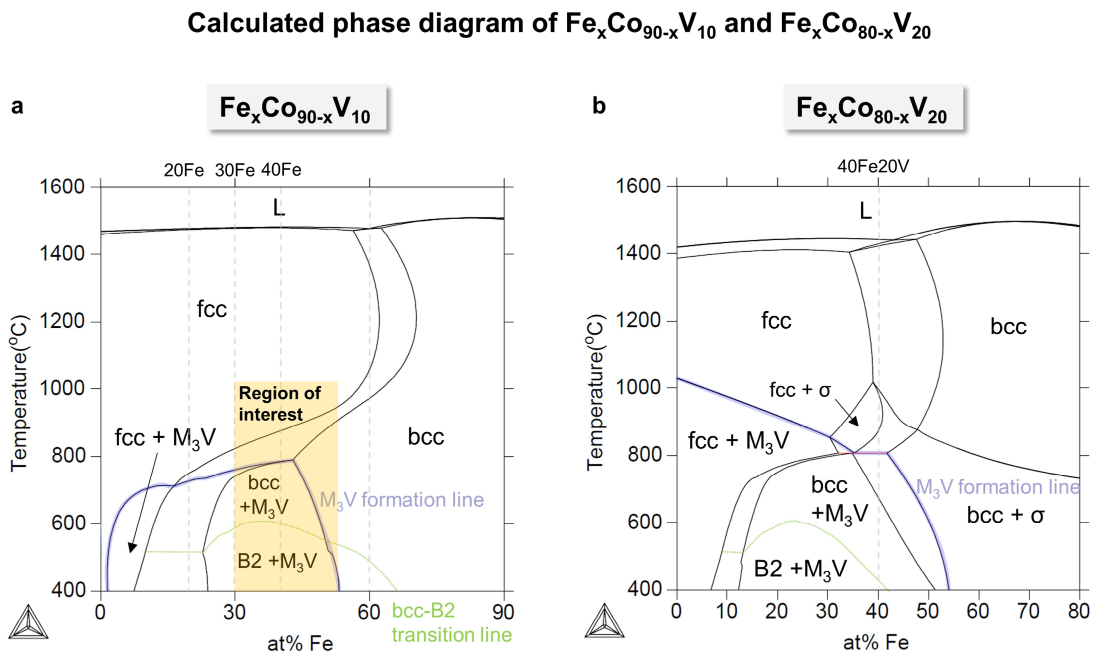

Supplementary Fig. 2. Thermodynamic calculation results (CALPHAD) of Fe–Co–V system with fixed V compositions. Phase diagrams at **a** $Fe_xCo_{90-x}V_{10}$ and **b** $Fe_xCo_{80-x}V_{20}$.

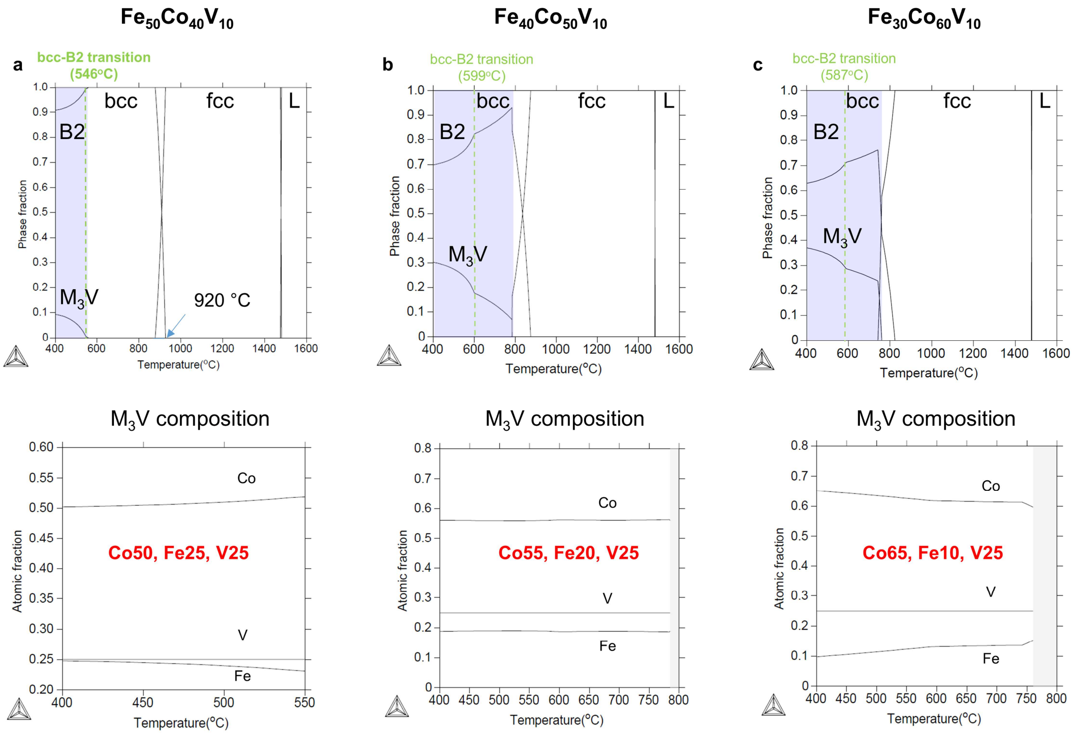

Supplementary Fig. 3. Thermodynamic calculation results of phase fraction vs. temperature and detailed M_3V composition. Results for alloy composition of **a** $Fe_{50}Co_{40}V_{10}$, **b** $Fe_{40}Co_{50}V_{10}$, and **c** $Fe_{30}Co_{60}V_{10}$.

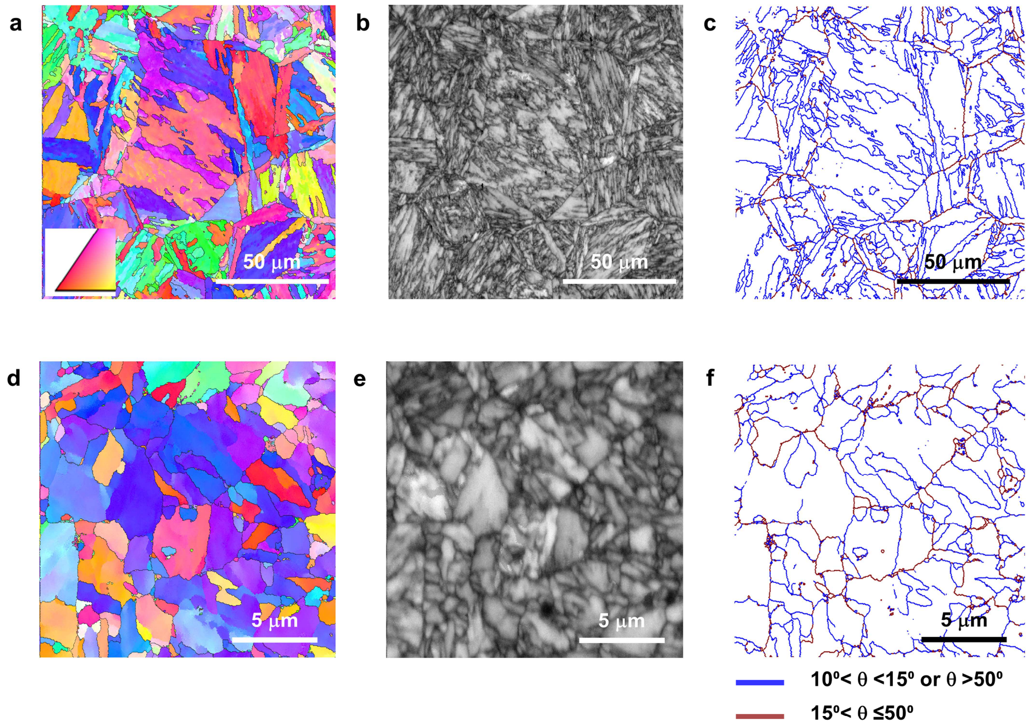

Supplementary Fig. 4. Characterisation of microstructure for solution annealed alloys. a–c EBSD IPF, IQ, boundary maps for martensite matrix transformed from coarse fcc grains (solution annealed at 1273 K (1000 ºC) for 60 min), **d–f** EBSD IPF, IQ, boundary maps for martensite matrix transformed from fine fcc grains (solution annealed at 1173 K (900 ºC) for 10 min).

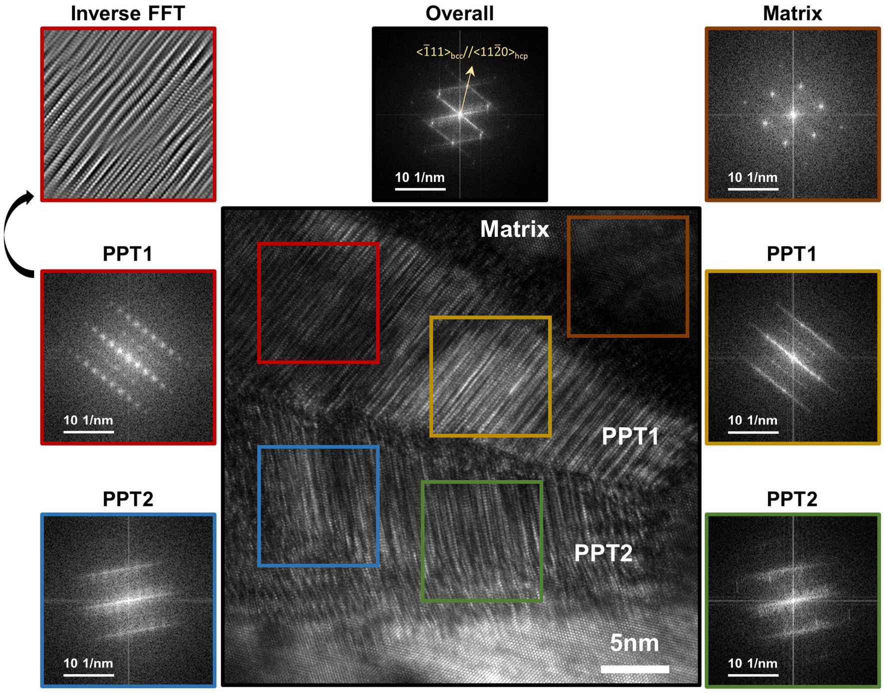

Supplementary Fig. 5. High-resolution transmission electron microscopy (HRTEM) image of two precipitates intersecting within the matrix. Fast Fourier-transformation (FFT) images for each spot are presented where the pattern clarity varies with site. Inverse FFT of the red box spot shows faulted structure of hP24 precipitate. The overall pattern indicates Burgers orientation relationship in $<\bar{1}11>_{bcc}//<11\bar{2}0>_{hcp}$ direction.

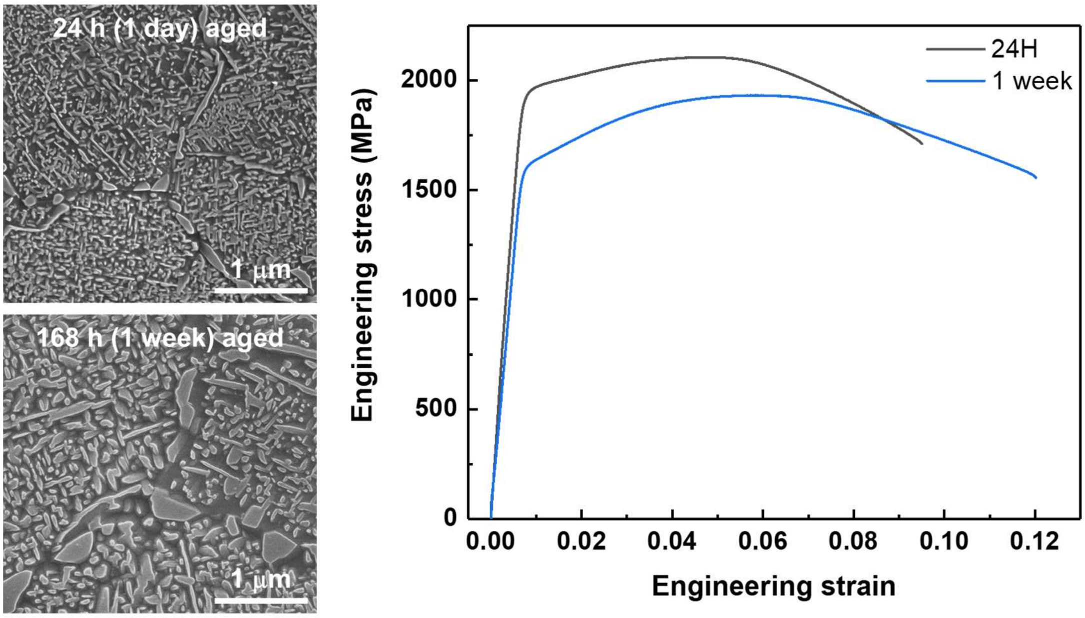

Supplementary Fig. 6. Comparison of microstructures and tensile properties for the alloys aged for 1 day (24H alloy) and 1 week. Prolonged aging treatment results in coarsening of precipitates, reducing strength but enhancing ductility. However, their semi-coherent interfaces enable the precipitates to maintain the nanometre size after further ageing up to 1 week.

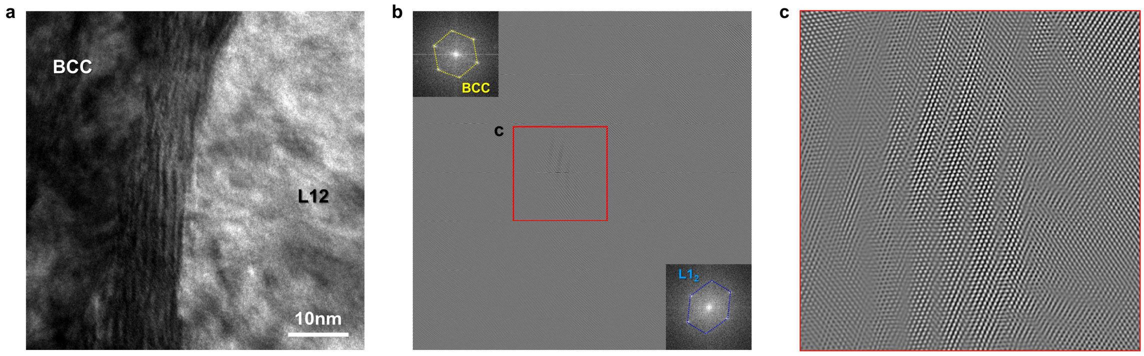

Supplementary Fig. 7. Phase interfaces between the matrix and grain-boundary $L1_2$ precipitates of the near-rational OR side. a BF image, **b** inverse fast Fourier transform (IFFT) spectra, and **c** enlarged IFFT spectra in **b**.

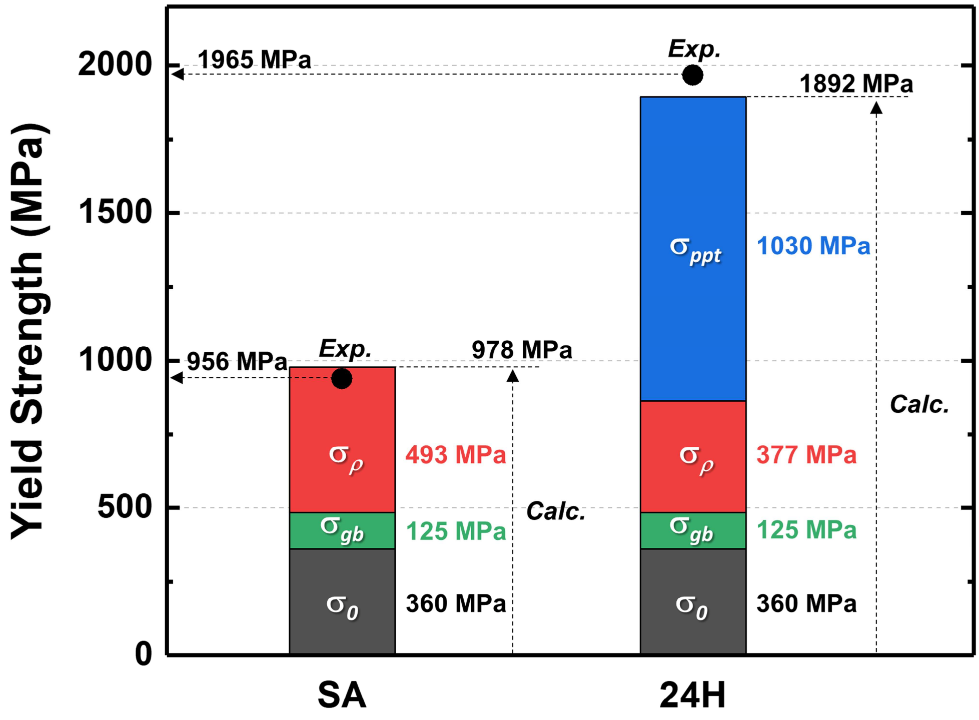

Supplementary Fig. 8. Calculated strengthening contributions (*Calc.*) to yield strength of SA and 24H alloys. Experimental values (*Exp.*) are indicated by black dots.

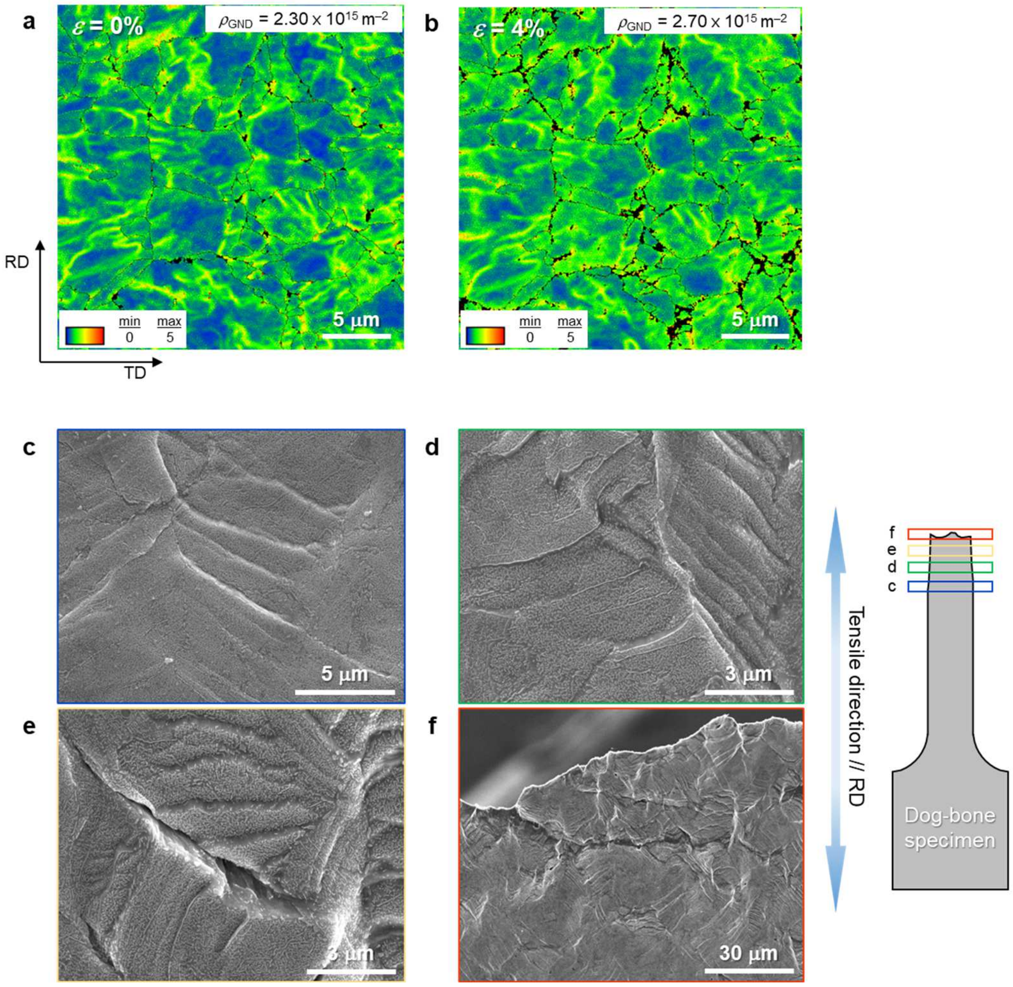

Supplementary Fig. 9. Microstructural characterisation of deformation evolution for 24H alloy. a,b EBSD kernel average misorientation (KAM) maps of the same area at different strain levels, **a** 0% and **b** 4%. The KAM maps reveal that the dislocation density increases homogeneously throughout the entire grains, where massive dislocation-precipitate interactions occur in nanometre scale as presented in Fig. 4b. **c–f** Observation of deformation bands evolution and failure through SEM images. **c** Parallel micro shear bands formed along boundaries and within grains over a wide range, **d** more parallel bands developed uniformly. **e** Severe shear bands lead to crack initiation at the boundary. **f** Macro-scale observation on the fractured region with the presence of numerous bands, which indicates uniform and homogeneous deformation occurred.

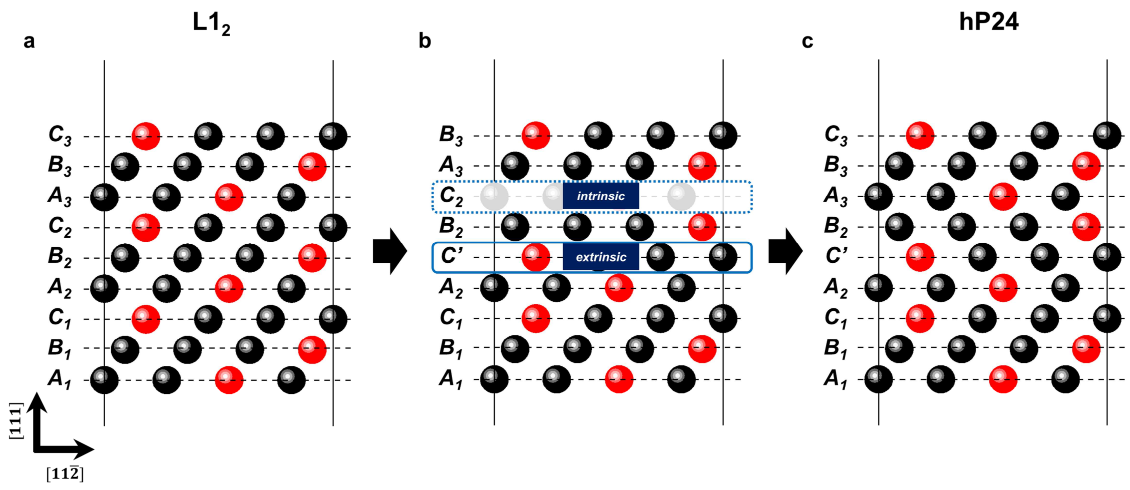

Supplementary Fig. 10. Schematic diagrams showing precipitation transformation sequences. **a** ordered fcc ($L1_2$) and **c** ordered hcp (hP24). **b** Superlattice extrinsic/intrinsic stacking fault pairs adding and removing C' and C_2 layers, respectively. (Note that subscripts are only for individualisation of each layer to visualise sequence change, where identical alphabets indicate equivalent layers at each state.)

Supplementary Table 1. Chemical composition of the matrix, $L1_2$, and hP24 (at%) for the 24H alloy

	Fe	Co	V
Matrix (bcc)	58.4	35.4	6.2
$L1_2$	21.8	53.8	24.4
hP24	23.7	51.7	24.6

Supplementary Table 2. Mechanical properties of solution-annealed and aged samples

	Yield Strength (MPa)	Tensile Strength (MPa)	Uniform Elongation (%)
SA	956 ± 20	1079 ± 16	2.0 ± 0.1
1H	1349 ± 10	1617 ± 7	7.7 ± 0.5
24H	1965 ± 24	2105 ± 17	4.0 ± 0.1

Supplementary Table 3. The formation energy (ΔE_f in eV/atom) the fully ordered structures (hP24, $L1_2$, and $D0_{19}$) at different compositions obtained by the present DFT calculation at 0 K. The reference states for each element are hcp Co, bcc Fe, and bcc V

Composition	hP24	$L1_2$	$D0_{19}$
Co_3V	–0.1714	–0.1660	–0.1637
Co_3Fe	0.0176	0.0131	0.0375
Fe_3V	–0.0405	–0.0559	–0.0046
Fe_3Co	0.1073	0.0993	0.1023
V_3Co	0.0740	0.0733	0.0868
V_3Fe	0.1417	0.3780	0.1372

Supplementary Table 4. Mole fraction (X_i) and number (N_i) of elements in the supercell used for DFT calculations of alloys with different compositions. Experimentally determined target compositions ($Exp._{L12}$ and $Exp._{hP24}$) are given in parentheses (Supplementary Table 1)

Alloy	X_{Co}	X_{Fe}	X_V	N_{Co}	N_{Fe}	N_V
Co_3V_1	0.75	0	0.25	36		12
$Exp._{L12}$	0.54167 (0.538)	0.20833 (0.218)	0.25 (0.244)	26	10	12
$Exp._{hP24}$	0.52083 (0.517)	0.22917 (0.237)	0.25 (0.246)	25	11	12
$Co_2Fe_1V_1$	0.5	0.25	0.25	24	12	12
$Co_{1.5}Fe_{1.5}V_1$	0.375	0.375	0.25	18	18	12
$Co_1Fe_2V_1$	0.25	0.5	0.25	12	24	12
Fe_3V_1	0	0.75	0.25		36	12

Supplementary References

1. Lee, B. J., Hwang, N. M. & Lee, H. M. Prediction of interface reaction products between Cu and various solder alloys by thermodynamic calculation. *Acta Mater.* **45**, 1867–1874 (1997).
2. Lee, B. J. Prediction of Ti/Al2O3 interface reaction products by diffusion simulation. *Acta Mater.* **45**, 3993–3999 (1997).
3. Villars, P. & Calvert, L. D. Pearson's Handbook of Crystallographic Data for Intermetallic Phases. (1986).
4. Lin, W., Xu, J. H. & Freeman, A. J. Electronic structure, cohesive properties, and phase stability of Ni3V, Co3V, and Fe3V. *Phys. Rev. B* **45**, 10863–10871 (1992).
5. Tosun, O. *et al.* Structural and magnetic properties of Co-V nanoparticles. *AIP Adv.* **9**, (2019).
6. Nagel, L. J., Fultz, B. & Robertson, J. L. Phase equilibria of Co3V. *J. Phase Equilibria* **18**, 21–23 (1997).
7. He, J. Y. *et al.* A precipitation-hardened high-entropy alloy with outstanding tensile properties. *Acta Mater.* **102**, 187–196 (2016).
8. Wang, Z., Baker, I., Guo, W. & Poplawsky, J. D. The effect of carbon on the microstructures, mechanical properties, and deformation mechanisms of thermo-mechanically treated Fe40.4Ni11.3Mn34.8Al7.5Cr6 high entropy alloys. *Acta Mater.* **126**, 346–360 (2017).
9. Zaddach, A. J., Scattergood, R. O. & Koch, C. C. Tensile properties of low-stacking fault energy high-entropy alloys. *Mater. Sci. Eng. A* **636**, 373–378 (2015).
10. Sohn, S. S. *et al.* Ultrastrong Medium-Entropy Single-Phase Alloys Designed via Severe Lattice Distortion. *Adv. Mater.* **31**, (2019).
11. Lei, Z. *et al.* Enhanced strength and ductility in a high-entropy alloy via ordered oxygen complexes. *Nature* **563**, 546–550 (2018).
12. Huang, H. *et al.* Phase-Transformation Ductilization of Brittle High-Entropy Alloys via Metastability Engineering. *Adv. Mater.* **29**, (2017).
13. Senkov, O. N. & Semiatin, S. L. Microstructure and properties of a refractory high-entropy alloy after cold working. *J. Alloys Compd.* **649**, 1110–1123 (2015).
14. Sheikh, S. *et al.* Alloy design for intrinsically ductile refractory high-entropy alloys. *J. Appl. Phys.* **120**, (2016).
15. Niu, S. *et al.* Strengthening of nanoprecipitations in an annealed Al0.5CoCrFeNi high entropy alloy. *Mater. Sci. Eng. A* **671**, 82–86 (2016).

16. Rao, J. C. *et al.* Secondary phases in AlxCoCrFeNi high-entropy alloys: An in-situ TEM heating study and thermodynamic appraisal. *Acta Mater.* **131**, 206–220 (2017).

17. Wang, Q. *et al.* A cuboidal B2 nanoprecipitation-enhanced body-centered-cubic alloy Al0.7CoCrFe2Ni with prominent tensile properties. *Scr. Mater.* **120**, 85–89 (2016).

18. He, J. Y. *et al.* Effects of Al addition on structural evolution and tensile properties of the FeCoNiCrMn high-entropy alloy system. *Acta Mater.* **62**, 105–113 (2014).

19. Li, D. *et al.* High-entropy Al0.3CoCrFeNi alloy fibers with high tensile strength and ductility at ambient and cryogenic temperatures. *Acta Mater.* **123**, 285–294 (2017).

20. Li, Z., Pradeep, K. G., Deng, Y., Raabe, D. & Tasan, C. C. Metastable high-entropy dual-phase alloys overcome the strength-ductility trade-off. *Nature* **534**, 227–230 (2016).

21. Yang, Y. *et al.* Bifunctional nanoprecipitates strengthen and ductilize a medium-entropy alloy. *Nature* **595**, 245–249 (2021).

22. Lu, Y. *et al.* Directly cast bulk eutectic and near-eutectic high entropy alloys with balanced strength and ductility in a wide temperature range. *Acta Mater.* **124**, 143–150 (2017).

23. Wang, Z. & Baker, I. Effects of annealing and thermo-mechanical treatment on the microstructures and mechanical properties of a carbon-doped FeNiMnAl multi-component alloy. *Mater. Sci. Eng. A* **693**, 101–110 (2017).

24. Liu, W. H. *et al.* Ductile CoCrFeNiMox high entropy alloys strengthened by hard intermetallic phases. *Acta Mater.* **116**, 332–342 (2016).

25. Yang, T. *et al.* Multicomponent intermetallic nanoparticles and superb mechanical behaviors of complex alloys. *Science* **362**, 933–937 (2018).

26. Shaysultanov, D. G. *et al.* Novel Fe36Mn21Cr18Ni15Al10high entropy alloy with bcc/B2 dual-phase structure. *J. Alloys Compd.* **705**, 756–763 (2017).

27. Schnitzer, R. *et al.* Influence of reverted austenite on static and dynamic mechanical properties of a PH 13-8 Mo maraging steel. *Mater. Sci. Eng. A* **527**, 2065–2070 (2010).

28. Jiang, S. H. *et al.* Strain hardening mediated by coherent nanoprecipitates in ultrahigh-strength steels. *Acta Mater.* **213**, (2021).

29. Sato, K. Improving the toughness of ultrahigh strength steel PhD thesis, Univ. California, Berkeley (2002).

30. Kim, Y. K., Kim, K. S., Song, Y. B., Park, J. H. & Lee, K. A. 2.47 GPa grade ultra-strong 15Co-12Ni secondary hardening steel with superior ductility and fracture toughness. *J. Mater. Sci. Technol.* **66**, 36–45 (2021).

31. Niu, M. *et al.* Precipitate evolution and strengthening behavior during aging process in a 2.5 GPa grade maraging steel. *Acta Mater.* **179**, 296–307 (2019).

32. Calcagnotto, M., Ponge, D., Demir, E., & Raabe, D. Orientation gradients and geometrically necessary dislocations in ultrafine grained dual-phase steels studied by 2D and 3D EBSD. *Mater. Sci. Eng. A* **527**, 2738–2746 (2010).
